# Poor education and urgent information need for emergency physicians about rare diseases in China

**DOI:** 10.1186/s13023-022-02354-1

**Published:** 2022-05-26

**Authors:** Lingli Zhou, Jun Xu, Jing Yang

**Affiliations:** Emergency Department, State Key Laboratory of Complex Severe and Rare Diseases, Peking Union Medical College Hospital, Chinese Academy of Medical Science and Peking Union Medical College, Beijing, China

**Keywords:** Rare diseases, Emergency physicians, Information needs, Rare disease

## Abstract

**Background:**

Rare diseases are serious and chronic diseases that affect no more than 1 person in 2000 (in European Union criteria). Patients suffering from RDs may come to the emergency department for life-threatening symptoms, such as acute aortic dissection, intracranial haemorrhage, and severe respiratory distress. Diagnostic delay of rare disease patients is common and often caused by low rare disease awareness among physicians. The main aim of this study was to investigate Chinese emergency physicians’ basic knowledge, information access and educational needs regarding rare diseases. An online questionnaire was completed by Chinese emergency physicians during January and March 2021.

**Methods and results:**

A total of 539 emergency physicians, including 200 females and 339 males, responded to the questionnaire-based study. More than half of the respondents were from Tertiary A hospitals and had engaged in medical clinical work for more than 10 years. Only 4.27% of respondents correctly estimated the prevalence of rare diseases. A few respondents knew the exact number of RDs in the first official list of rare diseases in 2018. A total of 98.5% of respondents rated their knowledge about rare diseases as minimal or insufficient. Most emergency physicians preferred to obtain information through search engines instead of specialized websites on rare diseases. A lack of practice guidelines or consensus was considered the most important reason for the diagnostic delay of RD. Practice guidelines or consensus and professional websites on rare diseases are urgently needed for emergency physicians.

**Conclusion:**

The investigation shows poor knowledge of rare diseases among emergency physicians. Practice guidelines and professional websites on rare diseases were the primary urgent needs for emergency physicians. Specialized RD courses should also be added to medical education.

## Introduction

Rare diseases (RDs) are diseases affect no more than 1 person in 2000, according to the European Union, and they are always serious, chronic, and often life-threatening conditions [1]. It has been estimated that 6–8% of the population will be affected by a rare disease [2], which means that there are at least 16.8 million RD patients in China. RDs are characterized by a severe condition, difficult diagnosis, high misdiagnosis rate and low curable rate, and the main patients are teenagers. Due to the high clinical complexity, patients with RDs often face lengthy diagnostic delays, with some families seeing between 3 and 10 different doctors and waiting > 5 years for a definitive diagnosis [3, 4].

Patients living with RDs face tremendous social and economic difficulties [5]. RDs need to be taken seriously and approached urgently. In recent years, China, like many other countries, is doing all it can to improve the health and wellbeing of patients with rare diseases. The National Rare Disease Registry System in China (https://www.nrdrs.org.cn) and the Rare Diseases Clinical Cohort Study were established in 2016 [6]. The National Health Commission declared the first list of rare diseases, including 121 rare diseases in 2018 and established the National Network of Rare Diseases (NNRD, https://www.nrdrs.org.cn) in 2019 for the diagnosis and treatment of rare diseases. The rare disease map has been designed for the management of RDs. Meanwhile, progress has been made in the limited availability of orphan drugs in China [7].

A wide range of health professionals were involved in diagnosing rare diseases, such as general practitioners, pediatricians, geneticists, and neurologists [3, 8]. General practitioners play an important role in the diagnosis and managements of rare disease patients [9, 10]. In China, general practice in its infancy and public health care is poor. Emergency physicians take on some of the responsibilities of general practitioners. Patients with rare diseases often seek medical assistance in emergency room(ER). Such as acute porphyria, patients usually come to emergency department for abdominal pain and constipation, with most of the patients diagnosed as having an intestinal obstruction and even accepted an enterectomy [11]. Patients with acquired hemophilia A present with repeated bleeding, delayed diagnosis and treatment endanger their lives [12]. The acute severity of RDs makes the clinical work of emergency physicians more challenging. Therefore, the knowledge of emergency physicians on RDs is important.

Previous studies have shown that health care professionals and medical students lack training and experience on RDs [2, 13]. Chinese health professionals are trying to train medical professionals to recognize rare disorders and make improvements in diagnosis and treatment [7]. The Guide for the Diagnosis and Treatment of RDs in Chinese was published in 2019 [14]. The first textbook in Chinese on rare diseases was published in Oct 2020 [15]. Recent research showed that only 5.3% of Chinese physicians were moderately aware or well aware of RDs [16]. The current situation of Chinese emergency physicians’ knowledge about rare diseases has not been reported. Our study aims to describe Chinese emergency physicians’ clinical practice regarding RDs, their information-seeking behaviours and their educational needs and preferences.

## Materials and methods

The study was conducted between January and March 2021 among emergency physicians in China. A separate online data-collection questionnaire was used for the survey through a web platform (www.wjx.cn), which permits centralized data collection and limits repetition by mobile numbers. The survey was conducted with a standard questionnaire that was constructed from themes based on a review of the literature and the study aim. It included 30 questions: 6 questions that addressed their demographic data and 24 items referring to respondents' knowledge of and attitudes towards RDs. The questionnaire consisted of four groups of questions. The first group included six questions referring to demographic information, including gender, career length, hospital level, technical titles, licensing province and whether the respondents had any advanced training experience in other hospitals.

The second group of thirteen questions concentrated on the emergency physicians’ knowledge of RDs. The respondents were asked about the incidence (in European Union criteria) and number of RDs. Progress has been made in policies defining RDs and limiting the availability of orphan drugs in China. Three questions referred to the current situation of RDs in China, including the estimated number of RD patients, whether there was any national registry system and the exact number of RDs in the first list of RDs in 2018. Four items were related to orphan drugs, the hereditary nature, and the most common age of onset of RDs. Baseline data and updated information on the prevalence of RDs in China are unavailable [17]. We listed 27 RDs that were relatively common in the clinic according to an analysis of hospitalization reports for RDs in China [18] and the experience of our emergency department. Therefore, all respondents were asked to indicate which RD they had encountered from that list of 27 RDs. The respondents were asked if they had first diagnosed any RD. The 140 respondents who had first diagnosed an RD were asked the number of RDs they had seen in their career and to indicate which RDs they first diagnosed from a list of twenty RDs; they could also indicate any RDs that they first diagnosed that were not on the list.

The third group included six questions related to the respondents' self-assessment and information access on RDs. The respondents were asked how they perceived their knowledge about RDs, whether they had any classes on RDs, how they learned about them, and which access route was the most effective. Two questions referred to which website they preferred for learning about RD and which professional websites on RDs they had used.

The last group of five questions referred to emergency physicians’ needs for RD information. The respondents were asked if they would like to learn more about RDs and the reasons delaying the diagnosis of RDs. The authors also wanted to know which aspect of RDs the emergency physicians preferred to learn more about. Finally, the respondents were asked which specialty requires more in-depth training on RDs and whether it is necessary to add an RD course to their medical school education. Ethics approval and research governance approval were obtained from Peking Union Medical College Hospital.

Statistical testing was performed using SPSS Statistics 25.0. The respondents were divided into different groups according to career length, hospital, and title. The rate of correct answers was the percent of respondents choosing the right answer in each group. The rate of correct results was analysed with the chi-squared test to reveal significant differences (p < 0.05).

## Results

### Demography

A total of 539 emergency physicians answered the questionnaire. All respondents were from China, including 27 of 34 Chinese provincial administrative regions, with the top five in Hubei (83, 15.4%), Liaoning (70, 12.99%), Guangxi (65, 12.06%), Henan (41, 7.61%), and Jiangxi (38, 7.05%). The demographic information of these respondents is listed in Table 1. Of 539 emergency physicians, 200 (37.11%) were female and 339 (62.89%) were male, while 244 (45.27%) had engaged in medical clinical work for more than 15 years. In China, a 3-tier system was established to recognize a hospital’s comprehensive abilities in medical care, education, and research [16]. Of 539 emergency physicians, 337 (62.52%) were from Tertiary A (the highest level) hospitals, while 65 (12.06%) were from Tertiary B hospitals and 137 (25.42%) were from secondary hospitals. The responders in this study included 99 (18.37%) residents, 209 (38.78%) attendings, 170 (31.54%) associate chief physicians and 61 (11.32%) chief physicians. A total of 331 (61.41%) respondents had advanced training experience in other hospitals.

**Table 1:** Demographic information of emergency physicians

Characteristic	Respondents (n = 539)	
	n	%
Gender		
Female	200	37.11%
Male	339	62.89%
Career length (years)		
< 5	64	11.87%
5 ~ 10	86	15.96%
10 ~ 15	145	26.90%
> 15	244	45.27%
Hospital		
Tertiary A	337	62.52%
Tertiary B	65	12.06%
Secondary	137	25.42%
Title		
Resident	99	18.37%
Attending	209	38.78%
Associate chief physician	170	31.54%
Chief physician	61	11.32%
Advanced training experience		
No	208	38.59%
Yes	331	61.41%

### Knowledge

**Fig. 1:**
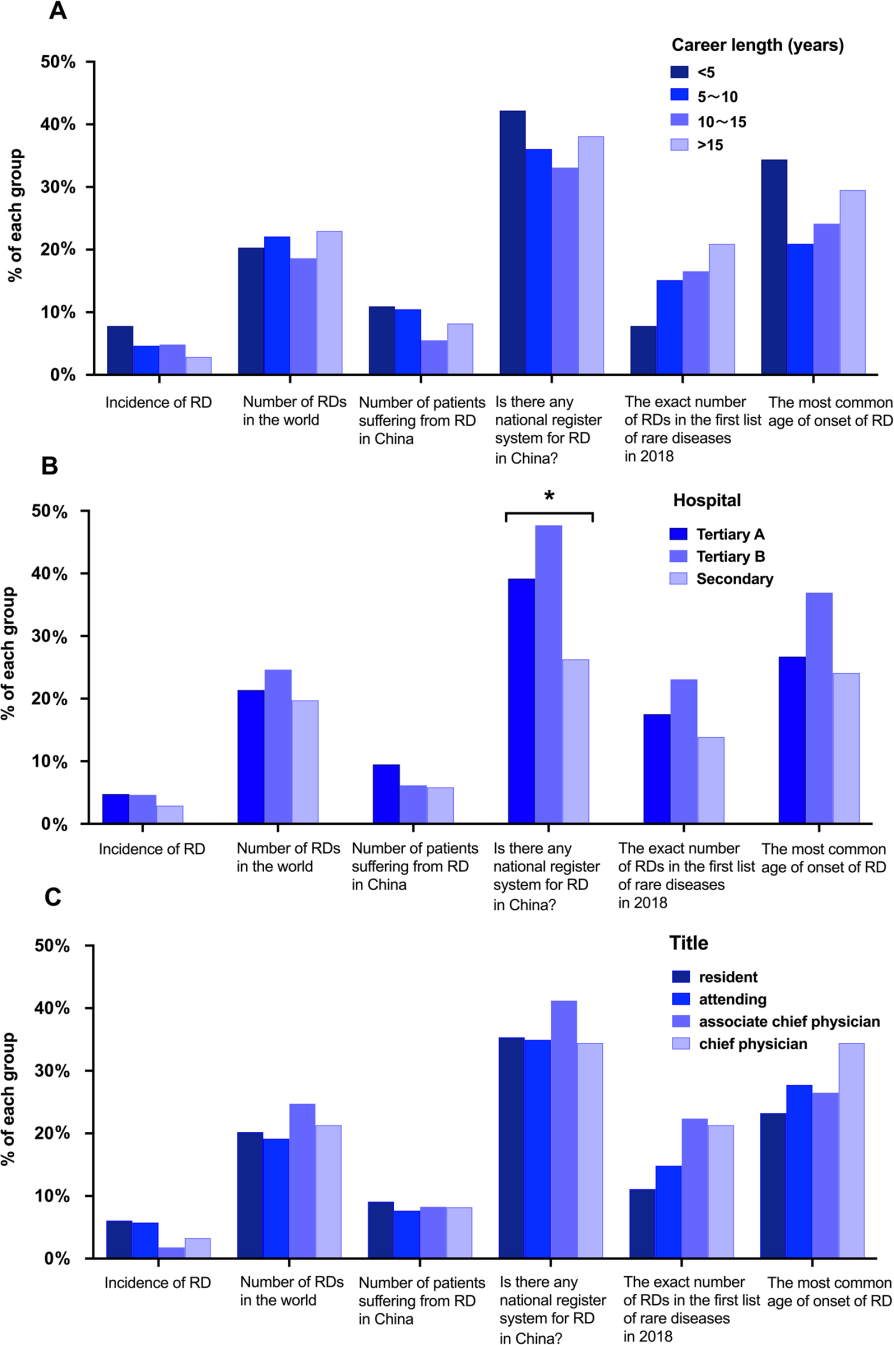
The knowledge of emergency physicians on six core questions on RDs. **A** The rate of correct answers in each career length group. **B** The rate of correct responses in each hospital level group. **C** The rate of correct responses in each title group. * p < 0.05, the respondents from secondary hospitals had a lower knowledge rate than those from Tertiary A or B hospitals (p < 0.05)

Respondents’ knowledge about RDs is shown in Table 2. Only 23 (4.27%) respondents correctly estimated the prevalence of RD (in European Union criteria), and 115 (21.34%) knew the number of RDs. Forty-four (8.16%) respondents were aware of the number of patients suffering from RD in China, while 199 (36.92%) knew that there was a national registry system for RDs in China. Ninety-three (17.25%) respondents knew the exact number of RDs in the first list of RDs in 2018, and 105 (19.48%) were aware of the percentage of RDs with orphan drugs. A total of 509 (94.43%) respondents knew that not all RDs were hereditary, but only 97 (18%) knew the percentage of hereditary diseases in RDs. A total of 147 (27.27%) respondents knew the most common age of onset of RDs. Only 140 (25.97%) respondents had first diagnosed an RD. Out of the 140 respondents, 98 (70%) had seen less than ten types of RD, 32 (22.86%) had seen ten to twenty types and only 10 (7.14%) had seen more than twenty types. For six core questions on RD, the rate of correct responses of respondents from different groups is shown in Fig. 1. The result of the chi-squared test shows that from different career length, hospital and title groups, the respondents have a similar knowledge rate of RD (p > 0.05). Only in the correctness of responses to “Is there any national register system for RD in China” did the respondents from secondary hospitals have a lower rate than those from Tertiary A or B hospitals (p < 0.05), and the rate of correct answers was not significantly different between Tertiary A and Tertiary B hospitals (p > 0.05).

**Table 2:** Emergency physicians’ knowledge of rare diseases

Items	n		%
Incidence of RD (in European Union criteria)			
< 1: 1000	66		12.24%
***< 1: 2000*** [19]	**23**		**4.27%**
< 1: 10,000	259		48.05%
I do not know	191		35.44%
Number of RDs in the world			
600–800	93		17.25%
***6000–8000*** [19]	**115**		**21.34%**
60,000–80,000	18		3.34%
I do not know	313		58.07%
Number of patients suffering from RD in China			
200,000	101		18.74%
2,000,000	112		20.78%
***20,000,000*** [7]	**44**		**8.16%**
200,000,000	0		0%
I do not know	282		52.32%
Is there any national register system for RD in China?			
***Yes*** [7]	**199**		**36.92%**
No	28		5.19%
I do not know	312		57.88%
The exact number of RDs in the first list of rare diseases in 2018			
***121*** [17]	**93**		**17.25%**
147	95		17.63%
I do not know	351		65.12%
The percent of RDs with orphan drugs			
0%	11		2.04%
***5%*** [5]	**105**		**19.48%**
10%	41		7.61%
20%	23		4.27%
50%	6		1.11%
I do not know	353		65.49%
Are all RDs hereditary?			
Yes	30		5.57%
***No*** [13]	**509**		**94.43%**
The percentage of hereditary diseases among RDs			
20%	100		18.55%
50%	72		13.36%
***80%*** [13]	**97**		**18%**
100%	2		0.37%
I do not know	268		49.72%
The most common age of onset of RD			
Neonatal period	45		8.35%
***Childhood*** [30]	**147**		**27.27%**
Adulthood	37		6.86%
Equal in all age groups	143		26.53%
I do not know	167		30.98%
Have you first diagnosed an RD in a patient?			
Yes	140		25.97%
No	399		74.03%
How many RDs have you seen in your career? *			
1 ~ 5	54		38.57%
5 ~ 10	44		31.43%
10 ~ 15	22		15.71%
15 ~ 20	10		7.14%
> 20	10		7.14%

Correct answers are indicated in bold and bolditalics

*This question was given to 140 respondents who first diagnosed an RD

The respondents were presented a list of twenty-seven RDs and asked to select those they had seen or first diagnosed (Table 3). For the 539 responding emergency physicians, the most common RDs they had seen were Marfan syndrome (49.17%), haemophilia (46.75%), idiopathic pulmonary fibrosis (46.01%), multiple sclerosis (41.37%) and idiopathic pulmonary arterial hypertension (40.63%). For the 140 emergency physicians who had first diagnosed RD, the most frequent RDs they first diagnosed were Marfan syndrome (38.57%), generalized myasthenia gravis (34.29%), idiopathic pulmonary arterial hypertension (30%), idiopathic pulmonary fibrosis (29.29%) and multiple sclerosis (24.29%).

**Table 3:** Which of the following rare diseases have been seen or first diagnosed?

Rare diseases	Which of the following rare diseases have you seen? (n = 539)		Which of the following rare diseases have you first diagnosed? (n = 140)	
Idiopathic pulmonary arterial hypertension	219	**40.63%**	42	**30%**
Idiopathic pulmonary fibrosis	248	**46.01%**	41	**29.29%**
IgG4-related disease	79	14.66%	16	11.43%
Lymphangioleiomyomatosis	45	8.35%	4	2.86%
Marfan syndrome	265	**49.17%**	54	**38.57%**
Multiple sclerosis	223	**41.37%**	34	**24.29%**
POMES syndrome	76	14.10%	12	8.57%
Porphyria	143	26.53%	24	17.14%
Albinism	204	37.85%	27	19.29%
Atypical haemolytic uraemic syndrome	49	9.09%	9	6.43%
Autoimmune encephalitis	157	29.13%	30	21.43%
Castleman disease	42	7.79%	3	2.14%
Congenital scoliosis	125	23.19%	19	13.57%
Diamond-Blackfan anaemia	38	7.05%	3	2.14%
Fanconi anaemia	35	6.49%	3	2.14%
Gaucher disease	42	7.79%	3	2.14%
Generalized myasthenia gravis	218	40.45%	48	**34.29%**
Glycogen storage disease	51	9.46%	2	1.43%
Haemophilia	252	**46.75%**	30	21.43%
Hepatolenticular degeneration	157	29.13%	32	22.86%
Pulmonary alveolar proteinosis	71	13.17%	16	11.43%
Amyotrophic lateral sclerosis	115	21.34%	–	–
Congenital myasthenic syndrome	63	11.69%	–	–
Huntington disease	59	10.95%	–	–
Idiopathic cardiomyopathy	61	11.32%	–	–
Langerhans cell histiocytosis	74	13.73%	–	–
Systemic sclerosis	173	32.10%	–	–
Others	10	1.86%	13	9.29%

The top five are indicated in bold

### Self-assessment and information access

**Fig. 2:**
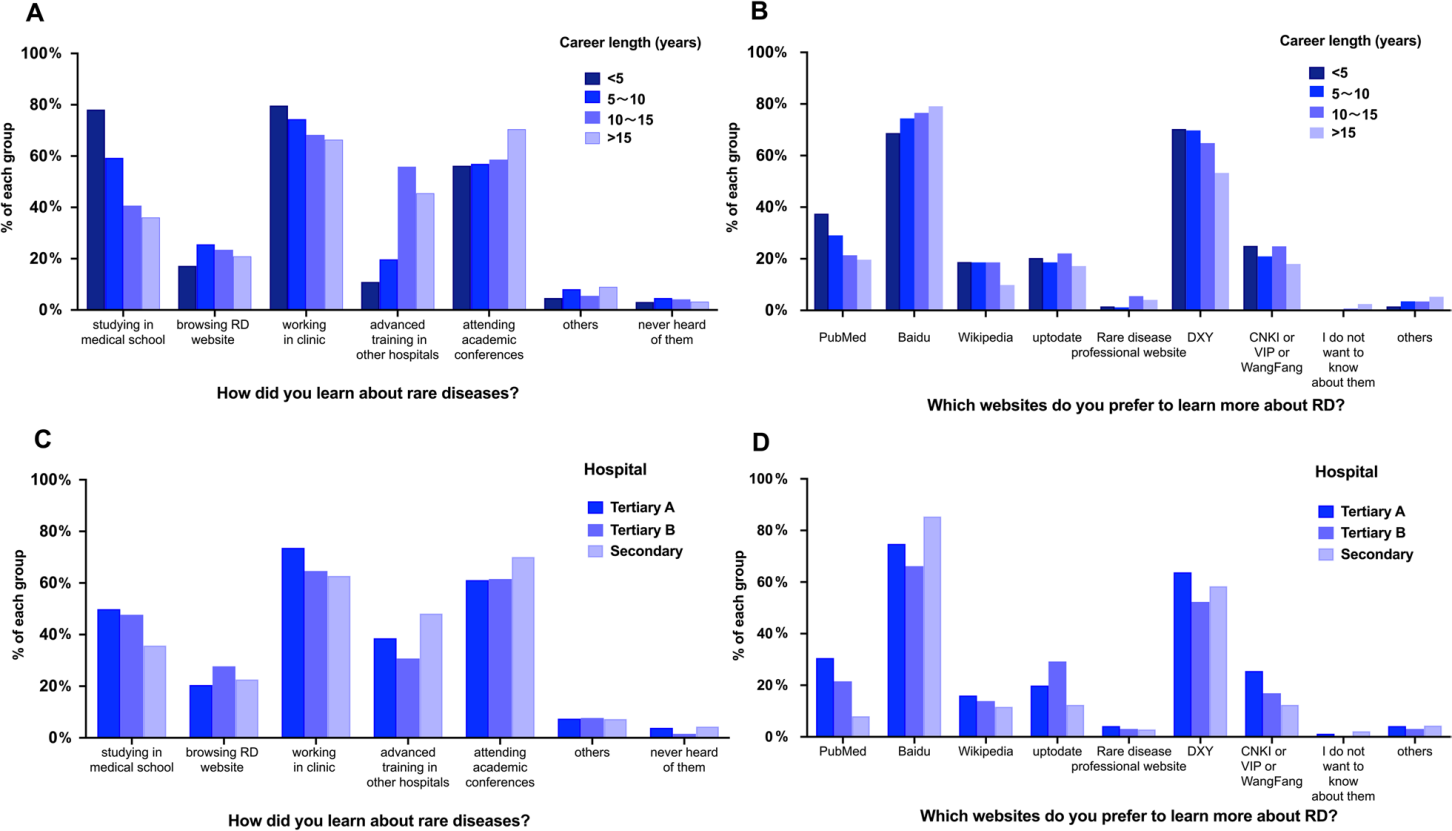
The information access of emergency physicians. **A** The distribution of information access in each career length group. **B** The distribution of preferential websites in each career length group. **C** The distribution of information access in each hospital group. **D** The distribution of preferential websites in each hospital group

The responses on self-assessment and information access on RDs are listed in Table 4. Only 1 (0.19%) respondent thought that he or she knew RDs well and 7 (1.30%) respondents considered themselves as having moderate knowledge, while 158 (29.31%) had insufficient knowledge and 373 (69.20%) had minimal knowledge of RDs. Most respondents (74.95%) did not take a course on rare diseases in their medical education. A total of 376 (69.76%) respondents learned about RDs in clinical work, and 232 (43.04%) thought it was the most effective way. A total of 412 (76.44%) respondents preferred Baidu for more information about RDs, and only 20 (3.71%) learned from professional websites on rare diseases. A total of 255 (47.31%) respondents had used the National Rare Disease Registry System of China. The answers to “How did you learn about RDs?” and “Which websites do you prefer to learn more about RD?” are shown in Fig. 2, and they were distinguished by different career lengths and hospital groups. With longer career length, the proportion of respondents learning about RDs by studying in medical school was lower. The respondents with longer career lengths had a lower preference for learning about RDs through PubMed. The respondents from secondary hospitals presented a lower proportion of having studied RDs in medical school or through academic websites, such as PubMed, CNKI, VIP and WanFang, but a high proportion of learning about RDs by advanced training in other hospitals and attending academic conferences.

**Table 4:** Self-assessment and information access of rare diseases

Items	N	%
How well do you know about rare diseases?		
Well	1	0.19%
Somewhat	7	1.30%
Insufficiently	158	29.31%
Minimally	373	69.20%
Was there any rare disease course in your medical education?		
Yes	84	15.58%
No	404	74.95%
I do not know	51	9.46%
How did you learn about rare diseases?		
Studying in medical school	248	46.01%
Browsing RD websites	118	21.89%
Working in clinic	376	69.76%
Advanced training in other hospitals	216	40.07%
Attending academic conferences	342	63.45%
Others	40	7.42%
Never heard of them	20	3.71%
Which access was most effective for you?		
Studying in medical school	29	5.38%
Surfing RD website	38	7.05%
Working in clinic	232	43.04%
Advanced training in other hospital	103	19.11%
Attending academic conferences	124	23.01%
Others	13	2.41%
Which websites do you prefer to use to learn more about RDs?		
PubMed	128	23.75%
Baidu	412	76.44%
Wikipedia	79	14.66%
Uptodate	103	19.11%
Rare disease specialist website	20	3.71%
DXY	329	61.04%
CNKI or VIP or WangFang	114	21.15%
I do not want to know about them	7	1.30%
Others	22	4.08%
Which professional websites on RD have you used?		
OMIM	71	13.17%
National Rare Disease Registry System of China	255	47.31%
Orphanet Portal for rare diseases	35	6.49%
ERAM or PEDAM	62	11.50%
Others	222	41.19%

### Information needs

**Fig. 3:**
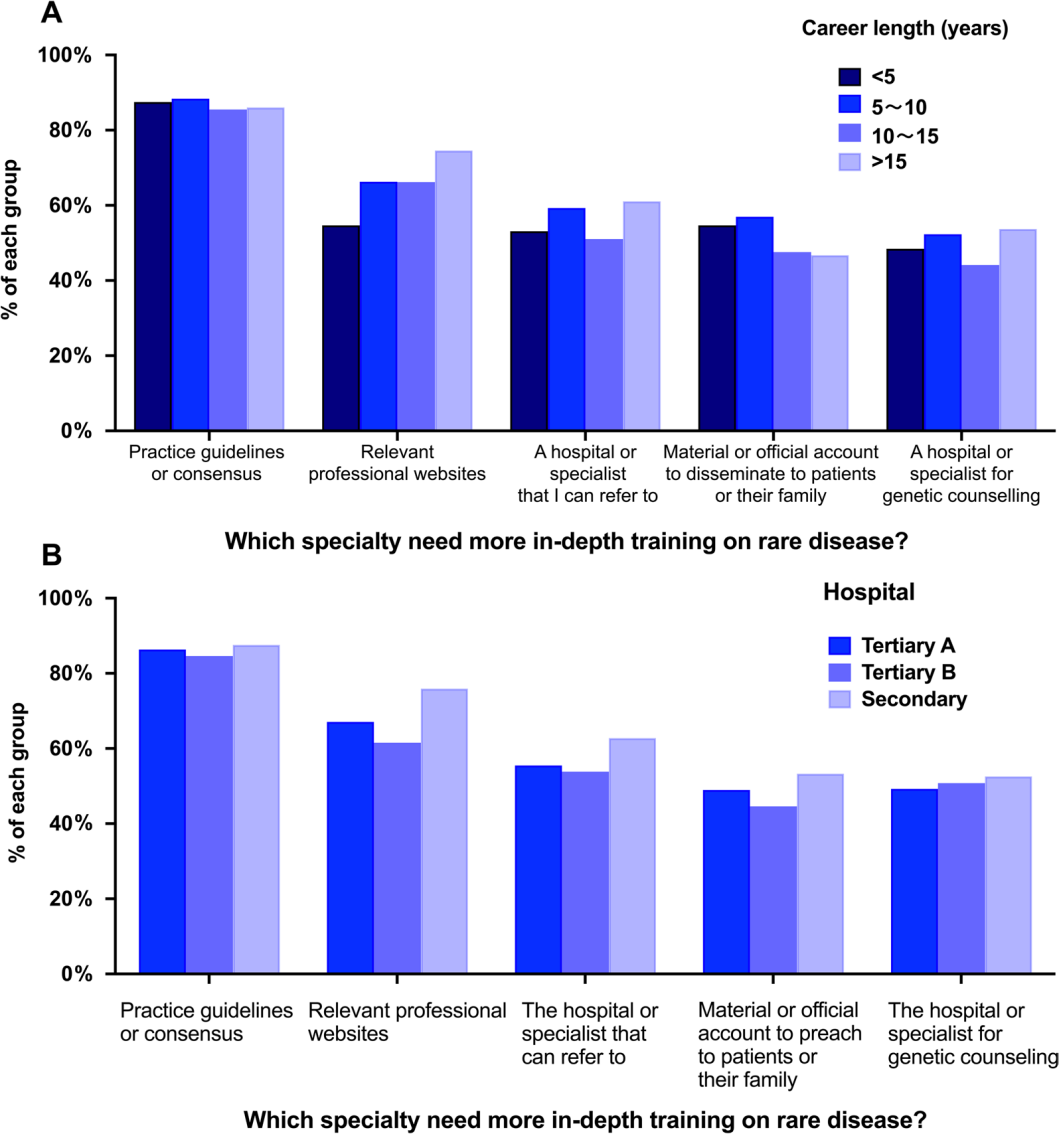
The information needs of emergency physicians. **A** The distribution of information needs in each career length group. **B** The distribution of information needs in each hospital group

Emergency physicians’ needs for RD information are shown in Table 5. Almost all respondents (92.21%) wanted to learn more about RD. A total of 450 (83.49%) respondents thought that a lack of practice guidelines or consensus delayed the diagnosis of rare disease. In addition, 466 (86.46%) preferred to learn more information about RDs by diagnostic guidelines or consensus. The distribution of information needs with the answer to “Which aspect of RDs do you prefer to learn more about?” is displayed by different career length and hospital groups (Fig. 3). Each group of career length or hospital had a similar proportion of information need. The respondents with more than 15 years of career experience or from secondary hospitals had a high proportion of needing relevant professional websites and hospitals or specialists that they could refer to. Half of the respondents (51.21%) thought that every physician, regardless of specialization, needed in-depth training on RDs. Moreover, 254 (47.12%) respondents thought that emergency physicians need more training on RDs, and 475 (88.13%) declared that it was necessary to add courses on rare diseases in medical school education.

**Table 5:** Emergency physicians’ needs for RD information

Items	N	%
Would you like to know more about rare diseases?		
Yes	497	92.21%
No	4	0.74%
I'm not sure	38	7.05%
The reasons delaying the diagnosis of rare disease		
Lack of practice guidelines or consensus	450	83.49%
Do not know how to find relevant professional websites	271	50.28%
Do not know a hospital or specialist that I can refer to	252	46.75%
Lack of material or official account to disseminate to patients or their family	225	41.74%
Do not know a hospital or specialist for genetic counselling	277	51.39%
Which aspect of rare diseases do you prefer to learn more about?		
Practice guidelines or consensus	466	86.46%
Relevant professional websites	370	68.65%
A hospital or specialist that I can refer to	308	57.14%
Material or official account to disseminate to patients or their family	267	49.54%
A hospital or specialist for genetic counselling	271	50.28%
Which specialty needs more in-depth training on rare diseases?		
Paediatrician	156	28.94%
General practitioner	212	39.33%
Neurologist	110	20.41%
Geneticist	186	34.51%
Physician	139	25.79%
Emergency physician	254	47.12%
Every physician regardless of specialization	276	51.21%
Other	9	1.67%
None	4	0.74%
Is it necessary to add a course on rare disease to your medical school education?		
Yes	475	88.13%
No	32	5.94%
Depends on the course content	32	5.94%

## Discussion

The most common definitions for what constitute a rare disease are the eligibility criteria used by drug regulatory agencies. Thus, different regions have different definitions. In the European Union, a disease is rare when it affects fewer than 1 in 2,000 people, whereas a rare disease is defined as a condition that affects fewer than 200,000 people in the United States [19]. In China, the first list of rare diseases was published in 2018, although there is no official definition for RDs. The incidence of each rare disease is not clear. The rare disease thematic maps are developing for management and in-depth research on RDs [20]. The questionnaire results indicated that emergency physicians have poor knowledge of RD and that an urgent need for RD information does exist. Although most respondents had been in medical practice for more than five years and half of them had advanced training experience, they lacked basic knowledge about RDs. Only 4.27% of respondents knew that a disease with an incidence lower than 1 in 2000 is defined as an RD in European Union criteria [19]. More than half of the respondents had no idea about the epidemiology, registry system or the first official list of RDs in China. Only eighteen percent of emergency physicians knew that 80% of RDs are hereditary, while 27.27% knew that the onset of RDs most frequently appears in childhood. The underestimation of the profile of rare diseases might indicate a low level of rare disease awareness.

Of our subjects, 98.5% rated their knowledge about RDs as minimal or insufficient, while Walkowiak et al. [21] reported that 94.6% of physicians in Poland perceived their knowledge of RDs as insufficient or very poor. Chinese physicians showed a similar result in a self-assessment on RDs: only 5.3% (12/224) had moderate or good awareness of RDs [16]. These results may be related to the low prevalence, complicated condition, and difficult diagnosis of RDs, which usually require multidisciplinary consultation.

To the best of our knowledge, this is the first research concentrated on emergency physicians’ education and information about RDs in China. A similarly poor cognitive level about RDs has also been reported in physicians [16, 22], surgeons [23], medical students [13], nurses and nursing students [24]. Meanwhile, from our research, only a quarter of respondents had experience with the first diagnosis of RDs (140, 25.97%). Although RDs are chronic diseases and are mostly diagnosed by specialists, patients suffering from RDs may come to the emergency department for life-threatening symptoms before the first diagnosis of RD. Our survey finds, the most frequent RDs first diagnosed by ER doctors were Marfan syndrome (38.57%), generalized myasthenia gravis (34.29%), idiopathic pulmonary arterial hypertension (30%), idiopathic pulmonary fibrosis (29.29%) and multiple sclerosis (24.29%). Since Marfan syndrome always not be diagnosed until an individual presents with an acute aortic dissection [25]. For haemophilia, intracranial haemorrhage can be a potentially deadly complication [26, 27]. Patients can present with severe respiratory distress when suffering from idiopathic pulmonary fibrosis, multiple sclerosis, idiopathic pulmonary arterial hypertension, or generalized myasthenia gravis [28, 29]. The above data also shows that in China, if emergency physicians lack knowledge on RDs, many RD patients with acute symptoms may miss their first chance for diagnosis.

Diagnostic delay is one of the most common problems encountered while caring for RD patients [30]. A survey of 462 Australian children living with RDs showed that 38% consulted ≥ 6 different doctors before receiving the correct diagnosis, 37% believed the diagnosis was delayed, and 27% initially received a wrong diagnosis [3]. Yan et al. [31] investigated the diagnosis experience of 1,010 adult RD patients in China. The results indicated that 72.97% of patients were misdiagnosed; they waited an average of 4.3 years and visited 2.97 hospitals before receiving the definitive diagnosis of an RD [31]. Many countries include UK highlighted the integral role of general practice, computer learning and diagnostic algorithms can aid Rds diagnosis and minimise clinician error [32].Although China has made great progress in the field of health care in the past decade, family medical services are still relatively inadequate, and emergency physicians deal with all patients in emergency cases and are responsible for further treatment and referrals, emergency physicians usually need to act as general practitioners. Therefore, it is important to improve the awareness of RDs among Chinese ER doctors, which can reduce the misdiagnosis of RDs and shorten the diagnosis delay time.

The routes to enhance the knowledge of RDs include medical school, clinical work, academic conferences and online. In our study, clinical work and academic conferences were thought to be the most common and effective forms of access for learning about rare diseases. We found that the respondents from secondary hospitals presented a lower proportion of having studied RDs in medical school or through academic websites but a high proportion of learning RDs by advanced training in other hospitals and attending academic conferences. The reason may be that secondary hospitals are closer to community hospitals, and there are fewer opportunities to acquire knowledge of RDs from daily work. Most emergency physicians also preferred to learn about RDs at academic conferences, where specialists would share vivid cases of RDs. As learning from clinical work and academic conferences are not systemic and has great uncertainty, through the years, technologies like functional imaging have changed our way of seeing and interpreting the RDs[33],enhance the ability of our emergency physicians to self-learn is very important.

Pauer et al. [34] indicated that the quality of information on the internet about RDs is low. The information about RDs from most popular websites our subjects prefer, such as Baidu (a common search engine in China, http://www.baidu.com/) and DXY (a platform for professional medical communication in China, http://www.dxy.cn/), are not professional. Only 3.71% of respondents preferred professional websites for learning more about RDs, and less than half had used the National Rare Disease Registry System of China (https://www.nrdrs.org.cn/). The National Network of RDs could increase the communication of physicians from different hospitals and improve the diagnosis and treatment of RDs [14, 35]. A complete professional RD website is conducive to diagnosis and in-depth study on RD in the clinical work of emergency physicians and can also serve as a sharing platform of academic conferences. Therefore, popularizing and simplifying the professional websites on rare diseases should be made a top priority.

In 2019, the first Chinese Guide for the Diagnosis and Treatment of RDs was published [14]. But our emergency physicians still regarded the lack of practice guidelines or consensus as the most important reason for the diagnostic delay of RD. For most of emergency physicians, this guide needs more popularization and interpretation, especially in awareness of critical conditions of RDs. A total of 47.12% of respondents considered that emergency physicians needed more in-depth training on RD, and 92.21% were willing to learn more. Only 15.58% of emergency physicians had taken RD courses in their medical education, a survey in medical students showed similarly poor education on RDs [13]. In undergraduate or postgraduate medical education, less than one-third of the physicians had received training in rare diseases in Spain [2]. Vandeborne et al. reported that most physicians had specific information needs regarding RDs in Belgium and that attending continuous training sessions on RDs could improve knowledge and awareness of RDs [22]. Academic and continuous medical education should focus on “red flags” to increase RD attentiveness and provide easy access to educational opportunities and information resources regarding RD [2, 22]. The first Chinese textbook on RDs was published in 2020 to fill the gap in the medical education of clinical students [15]. E-learning programs and courses for RDs should also be organized [21].We plan to develop RD training courses for urgent and critical conditions of RDs based on the results of this survey. As the internet is the most convenient access of information, publishing online practice guidelines and popularizing professional websites on RDs are also urgent needs that could be effective for emergency physicians.

## Limitations

This is a questionnaire-based study on emergency physicians. Some items might be inaccurate, as they were answered on account of the respondents’ memory, and objective medical records were not used. The demographic distribution of respondents may be different from that of all emergency physicians. The emergency physicians who responded to this questionnaire may be more concerned with RD than those who refused to participate. Thus, this study was limited by underlying inaccurate responses to questionnaires and possible selection bias.

## Conclusion

In conclusion, the investigation shows poor knowledge and urgent information needs regarding RD in emergency physicians. It is imperative for the public health system to improve the training on RDs of emergency physicians, including by adding specialized RD courses in medical education, providing practice guidelines, and popularizing professional websites on RDs.

## Data Availability

The data used and/or analysed to support the results of the current study are available from the corresponding author upon reasonable request.
